# Challenging complications of treatment – human herpes virus 6 encephalitis and pneumonitis in a patient undergoing autologous stem cell transplantation for relapsed Hodgkin's disease: a case report

**DOI:** 10.1186/1743-422X-6-111

**Published:** 2009-07-20

**Authors:** Martin Bommer, Sandra Pauls, Jochen Greiner

**Affiliations:** 1 Department of Internal Medicine III – Hematology/Oncology, University of Ulm, Ulm, Germany; 2 Department of Diagnostic and Interventional Radiology, University of Ulm, Ulm, Germany

## Abstract

**Background:**

Reactivation of human herpesvirus 6 (HHV-6) occurs frequently in patients after allogeneic stem cell transplantation and is associated with bone-marrow suppression, enteritis, pneumonitis, pericarditis and also encephalitis. After autologous stem cell transplantation or intensive polychemotherapy HHV-6 reactivation is rarely reported.

**Case report:**

This case demonstrates a severe symptomatic HHV-6 infection with encephalitis and pneumonitis after autologous stem cell transplantation of a patient with relapsed Hodgkin's disease.

**Conclusion:**

Careful diagnostic work up in patients with severe complications after autologous stem cell transplantation is mandatory to identify uncommon infections.

## Background

Viruses that belong to the herpes group such as HSV1/2, HHV6 and CMV are known to reactivate after intensive immunosuppressive treatment. In patients receiving allogeneic stem cell transplantation reactivations are frequently reported [1-3]. Several reports showed a broad variety of clinical manifestation, ranging from asymptomatic reactivation, delayed hematopoietic recovery up to severe systemic infection with pneumonia and encephalitis [4-8]. Reports with severe HHV6 associated complications are limited to patients receiving allogeneic transplantation or – in the autologous setting – to paediatric patients [9]. Reports of severe complications caused by HHV6 after autologous stem cell transplantation or after intensive chemotherapeutic treatments are very rare due to infrequent events, but maybe also seldom due to lack of specific diagnostic approaches.

Diagnosis of HHV6 Infection remains basically PCR-based with detection of viral DNA in blood, cerebrospinal fluid and bronchoalveolar lavage [10]. Recently evidence for integration of HHV6B-DNA in leukocytes without any clinical relevance was reported, arousing doubts about unjustified diagnosis and treatment of HHV6 infection in transplant recipients[11].

## Case presentation

**Figure 1:**
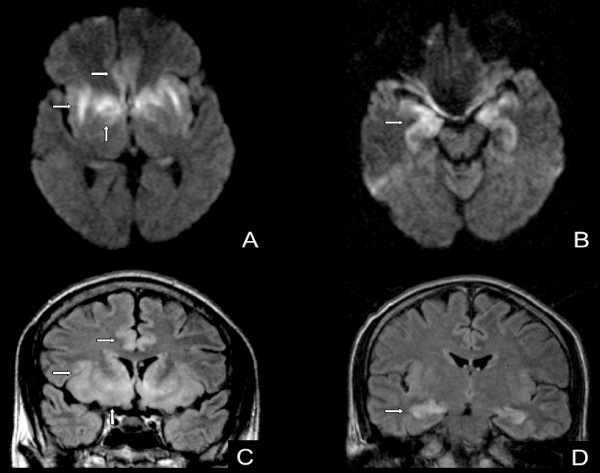
**MRI of the brain**: Typically bilateral and asymmetric encephalitis of the limbic system. Diffusion weighted images (A + B; axial view): restricted diffusion (hyperintense signal) in the cingulate gyri, insula, and temporal lobes (arrows). FLAIR (C + D; coronal view) sequences: hyperintense swollen cortex and subcortical white matter (arrows) in the medial temporal lobes and cingulate gyri.

**Figure 2:**
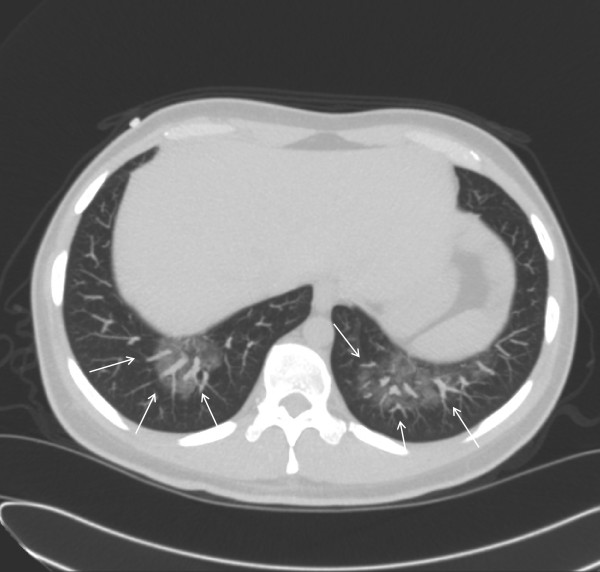
**Chest-CT**: Bilateral interstitial infiltrates.

A twenty-eight years old male was admitted to our hospital with relapsed Hodgkin's disease. He had received four cycles of ABV and involved field radiation. Seven months later the lymphoma relapsed and two cycles of Dexa-BEAM with stem cell harvest were applied. We performed high-dose chemotherapy according to the BEAM protocol. On day twelve after stem cell reinfusion he developed mental disturbance and convulsive status. First MRI imaging of the brain showed no abnormality. Lumbar puncture was done. Cell count of the cerebrospinal fluid (CSF) was > 300 μl with predominant lymphocytes. Polymerase chain reaction (PCR) test was positive for HHV6b DNA and negative for HSV 1, HSV2, EBV, CMV and enteroviridae. CT-Scan of the chest revealed diffuse bilateral interstitial pneumonia (Figure 1). Bronchoalveolar lavage was positive for HHV6b too and negative for Adenovirus, Influenza, Parainfluenza, Respiratory syncytial virus and Legionella pneumophilia. Immediate treatment with foscarnet and intravenous immunoglobulin was initiated. A second MRI of the brain two days later (Figure 2) showed diffuse inflammation compatible with herpes encephalitis. Initially the situation deteriorated due to respiratory failure and bilateral jugular vein thrombosis. Foscarnet – treatment was continued until day 44. Cerebrospinal fluid and peripheral blood were both negative for HHV6b using PCR. An oral therapy with valganciclovir was started and continued for another six weeks. The patient could be discharged from the hospital on day 48 after autologous stem cell transplantation. He recovered almost completely from his encephalitis, but unfortunately his lymphoma relapsed within nine months.

## Conclusion

We report an extremely uncommon infectious complication in a patient with relapsed Hodgkin's disease. Whereas asymptomatic HHV6 reactivation is frequently reported in patients after allogeneic stem cell transplantation, severe disease is rare in patients after autologous stem cell transplantation. Nevertheless, in patients with severe complications of infections after autologous stem cell transplantation or intensive chemotherapeutic treatment, HHV-6 detection should be included into the diagnostic work-up for these patients and longitudinal observational clinical studies have to be performed to examine the frequency of clinically relevant HHV-6 infections in these patient cohorts.

## Consent statement

Written informed consent was obtained from the patient for publication of this case report and accompanying images. A copy of the written consent is available for review by the Editor-in-Chief of this journal.

## Competing interests

The authors declare that they have no competing interests.

## Authors' contributions

GJ and BM were responsible for the patients care, PS interpreted the chest-CT and the MRI and added the figures, BM wrote the paper and all authors read and approved the final manuscript.

## Authors' information

G.J. and B.M. are attending physicians in the department of hematology and oncology of the University of Ulm

P.S. is attending physician in the department of Diagnostic and Interventional Radiology of the University of Ulm
